# Expansion of inflammatory monocytes in periphery and infiltrated into thyroid tissue in Graves’ disease

**DOI:** 10.1038/s41598-021-92737-4

**Published:** 2021-06-29

**Authors:** Xinxin Chen, Yanqiu Wang, Yicheng Qi, Jiqi Yan, Fengjiao Huang, Mengxi Zhou, Weiqing Wang, Guang Ning, Yulin Zhou, Shu Wang

**Affiliations:** 1 Department of Endocrinology, Shanghai Clinical Center for Endocrine and Metabolic Diseases, Shanghai Institute of Endocrine and Metabolic Diseases, Ruijin Hospital, Shanghai Jiao Tong University Medical School, 197 Ruijin 2nd Road, Shanghai, 200025 People’s Republic of China; 2 Department of Endocrinology, Suzhou Municipal Hospital, Nanjing Medical University, 26 Daoqian Road, Suzhou, 215002 People’s Republic of China; 3 Department of Thyroid Surgery, Ruijin Hospital, Shanghai Jiao Tong University Medical School, 197 Ruijin 2nd Road, Shanghai, 200025 People’s Republic of China

**Keywords:** Thyroid diseases, Autoimmunity

## Abstract

Monocytes are important mediators of immune system and are reported to be altered in autoimmune disorders. Little is known about the pathological role of monocytes in Graves’ disease (GD). Thus, we investigated monocytes in periphery and thyroid tissue in GD. Untreated GD patients were enrolled and followed up until remission. Monocytes were significantly increased and positively correlated with anti-thyrotropin receptor antibody (TRAb) in untreated GD (*r*_*counts*_ = 0.269, *P* < 0.001; *r*_*percentage*_ = 0.338, *P* < 0.001). Flow cytometry showed CD14++ CD16+ monocytes were increased and CD14++ CD16- monocytes were decreased in untreated GD (both *P* < 0.001). Skewed monocyte subsets were recovered in GD with remission. Serum B cell-activating factor (BAFF) was positively correlated with TRAb (*r* = 0.384 and *P* = 0.001). CD14++ CD16+ monocytes expressed higher level of BAFF in untreated GD (*P* < 0.05). The frequency of CD14+ monocytes and CD14+ CD16+ monocytes were significantly higher in GD thyroid tissue than in normal thyroid tissue (both *P* < 0.001). Our study suggested CD14++ CD16+ monocytes were significantly expanded and involved in the production of TRAb via secreting a higher level of BAFF in periphery. Besides, monocytes infiltrated into thyroid tissue and thus could serve as an important participant in GD pathogenesis.

## Introduction

Graves’ disease (GD) is an organ-specific autoimmune disease characterized by the presence of anti-thyrotropin receptor antibodies (TRAbs) in the circulation^1^. TRAbs can bind to thyroid stimulating receptor (TSHR) expressed on thyroid follicular cells and stimulate thyroid follicular cells to produce and release excessive amounts of thyroid hormones^2^. GD is a multifactorial disease caused by the interplay among susceptibility genes, environmental factors and immunity^1^. Immunity plays a central role in the production of TRAbs. Many studies validated the activated T/B cells both in periphery and in the thyroid gland in GD patients^1,3–5^. In contrast to the well-defined roles of T/B cells in the pathophysiology of GD, little is known about that of monocytes. Monocytes are key components of the innate immune system. In the steady state, monocytes circulate in the blood and increase and infiltrate into tissue during inflammation. By presenting antigens to T cells and secreting inflammatory cytokines such as tumor necrosis factor-α (TNF-α), interleukin-2 (IL-2), IL-4, IL-6, B cells activator factor (BAFF), monocytes are key regulators that promote the activation of T and B cells^6^. In autoimmune disease, monocytes and these specific cytokines may participate in initiating and propagating immune responses^7^.

On the basis of CD14/CD16 expression, human monocytes can be classified into three subsets: CD14++ CD16- (classical monocytes), CD14++ CD16+ (intermediate monocytes), and CD14+ CD16+ (nonclassical monocytes) ^8–9^. CD14++ CD16- monocytes account for the majority of circulating monocytes in the steady state, and they are not the predominant cytokine-producing monocytes in humans. The latter two monocyte subsets (CD14++ CD16+ and CD14+ CD16 +) are also known as inflammatory monocytes. Alteration in monocyte subsets is associated with a plethora of autoimmune diseases, such as rheumatoid arthritis, Crohn’s disease, multiple sclerosis, and systemic lupus erythematosus^10–13^. In multiple sclerosis, the expansion of classical (CD14++ CD16-) and nonclassical monocytes (CD14+ CD16 +) are sensitive markers of inflammation and could help to identify relapsing–remitting multiple sclerosis earlier^14^. However, very few studies have focused on the subsets of monocytes in GD. The first report analyzing the phenotypic characteristics of monocytes was published in 1998 and found that CD14+ CD16+ DR^high^ monocytes were abundant and correlated with CD4 + CD45 + RO + memory lymphocytes in GD patients^15^. The increased CD14+ CD16+ DR^high^ monocytes were thought to be involved in the repeated presentation of autoantigens or mimetic antigens to helpers T cells in GD.

Originating from bone marrow-derived progenitor cells, monocytes are precursor cells that may give rise to macrophages and dendritic cells in tissues. Monocytes have been found at the site of inflammation in autoimmune diseases, such as atopic dermatitis, psoriasis, and rheumatoid arthritis^16–18^. The infiltration of monocytes into locally inflamed tissue may play important roles in maintaining and amplifying the immune response. However, little is known about the presence of monocytes in the local thyroid tissue in GD patients.

Concerning the limited exploration of monocytes in GD, we aimed to explore the pathological roles of monocytes in the peripheral blood and thyroid tissue in GD patients in this study. The correlations of monocytes and clinical parameters were analyzed in GD patients before and after antithyroid treatment. In addition, the subsets of monocytes, specific cytokines and the presence of monocytes in local tissue were also evaluated in our study.

## Methods

### Particpants

Patients with new-onset, untreated GD (untreated GD, uGD) were recruited from the outpatient department of Ruijin Hospital affiliated to Shanghai Jiao Tong University Medical School. GD was diagnosed based on clinical symptoms and laboratory findings according to the 2016 American Thyroid Association (ATA) guidelines for the diagnosis and management of hyperthyroidism and other causes of thyrotoxicosis^19^. The clinical symptoms included heat intolerance, fatigue, increased appetite, increased sweating, weight loss, muscle weakness, tremors, and diffusely enlarged thyroid glands. The laboratory results included increased serum concentrations of free thyroxine (FT4) and/or free triiodothyronine (FT3), decreased basal thyroid-stimulating hormone (TSH) levels, and TRAb positivity. All GD patients in our study were taking antithyroid drugs (ATDs) and received regular follow-up in the outpatient department of Ruijin Hospital. ATDs, including Methimazole and Propylthiouracil, mainly through reducing the synthesis of thyroid hormones or inhibiting the conversion of T4 to T3. The exclusion criteria included age < 15 years; taking any medical regimen affecting thyroid function such as thyroid hormones, glucocorticoids, dopamine, or amiodarone; moderate to severe ophthalmopathy; a history of radioisotope therapy for Graves’ disease; pregnancy or lactation; malignant tumor; multiple organ disease and other conditions that prevented participation in the follow-up procedure. Age- and sex-matched healthy controls (HCs) were also enrolled in our study.

Patients with normal thyroid function but TRAb positivity were defined as having euthyroid (eGD). GD patients in remission were defined as those patients with maintenance of normal thyroid function and TRAb-negative GD (nGD) after ATD withdrawal for at least 1 year according to the 2016 ATA guidelines^19^. In our study, a total of 83 uGD patients have completed the follow-up procedure until remission. This study was conducted in accordance with the Declaration of Helsinki and was approved by the institutional review board of Ruijin Hospital affiliated to Shanghai Jiao Tong University Medical School. Written informed consent was obtained from each participant.

### Laboratory analysis

Routine testing of the peripheral venous blood for white blood cells (including total number of white blood cells, monocytes, lymphocytes, and neutrophils) was performed on all participants in our study. The test was analyzed by Sysmex XN-9000 (Sysmex Corporation, Kobe, Japan) blood cell analyzer by flow cytometry using a semiconductor laser. FT3, FT4, and TSH levels were measured using automated chemiluminescent immunoassays (ARCHITECT i2000SR, Abbott Laboratories, Chicago, IL). TRAb level was measured using electrochemiluminescence immunoassays (Cobas 601 analyzer, Roche Diagnostics). The laboratory reference ranges provided by the manufacturer were used in this study, as follows: TSH 0.35–4.94 μIU/mL, FT4 9.01–19.04 pmol/L, FT3 2.63–5.70 pmol/L, and TRAb < 1.75 IU/L. All tests were calibrated and measured by experienced technicians in Clinical Diagnostic Laboratory in Ruijin Hospital.

### Cell isolation and culture

Peripheral blood samples were collected from GD patients and healthy controls. The peripheral blood mononuclear cells (PBMCs) were isolated via Ficoll-Hypaque density gradient centrifugation (Sigma Aldrich, St. Louis, MO, USA). Monocytes were purified from Ficoll-enriched PBMCs using human CD14 microbeads (Stemcell Technologies, Canada). The purification of monocytes was carried out according to the manufacturer’s instructions. Freshly isolated PBMCs or CD14+ monocytes were cultured in RMPI 1640 supplemented with 10% fetal bovine serum, 1% penicillin at 37 °C and in a 5% CO_2_ atmosphere.

### Flow cytometry

PBMCs were rinsed twice with staining buffer and re-suspended in staining buffer. Then, the cells were incubated with specific extracellular antibodies for 30 min. The following monoclonal antibodies were used for monocyte phenotypic characterization: CD14-FITC and CD16-PE (BioLegend, San Diego, CA, USA). After fixation and permeabilization using Cytofix/Cytoperm Kit (BD, Biosciences), cells were stained with anti-BAFF-APC (BioLegend, San Diego, CA, USA) for 30 min to investigate the expression of BAFF on monocytes subsets. After staining with monoclonal antibodies, cells were washed twice with staining buffer and re-suspended in staining buffer. Appropriate isotype controls were used (showed in supplemental Fig. S1). All samples were acquired using LSRFortessa X-2 (BD bioscience). The data were analyzed using FlowJo software (Tree Star, Inc., San Carlos, CA, USA), and the mean fluorescence intensity (MFI) was adopted for the expression of BAFF on monocytes.

### RNA extraction and quantitative real-time polymerase chain reaction (PCR)

Total RNA was isolated from monocytes using trizol reagent (Invitrogen) according to the manufacturer’s protocol. In total, 1 μg of RNA was converted into first-strand cDNA with the first-strand cDNA Synthesis Kit (Promega, Madison, WI, the USA) according to the manufacturer’s instructions. Real-time quantitative PCR was performed using SYBR Master Mix (Takara, Shiga, Japan) on a LightCycler 480 system (Roche, Pleasanton, CA, USA). Human *GAPDH* was used as an endogenous control for sample normalization. The results are presented as the fold expression relative to that of *GAPDH*. The following PCR primers were used: for human *GAPDH*, forward 5′-TGATGACATCAAGAAGGTGGTGAAG-3′ and reverse 5′-TCCTTGGAGGCCATGTGGGCCAT-3′; for human BAFF, forward 5′- ACAGAAAGGGAGCAGTCACG-3′: and reverse 5′-GACAGAGGGGCTTTCCTTCC-3′.

### Analysis of serum B cell-activating factor (BAFF) levels by ELISA

BAFF levels were measured in the serum of 56 uGD patients, 12 nGD patients and 14 HCs using commercially available ELISA kits according to the manufacturer’s instructions. The ELISA kit for human BAFF cytokine was purchased from R&D Systems. The minimum detectable dose of each kit ranged from 62.5 to 4000 pg/mL, with intra- and inter-coefficients of variation of < 10%.

### Immunofluorescence

Thyroid tissues were obtained from six patients with GD and three patients with thyroid nodules (no Graves’ disease history and TRAb < 1.75 IU/L) in the department of Thyroid Surgery at Ruijin Hospital. The thyroid tissues were fixed with formalin and embedded with paraffin. Serial tissue sections (5 μm) were dewaxed and stained for immunofluorescence analysis. The thyroid tissue sections were blocked with 10% normal goat serum (Vector Laboratories) for 30 min. Primary antibodies were incubated overnight at 4 °C and amplified with the appropriate secondary antibodies. The primary and secondary antibodies used in this study are shown in Supplemental Table S1. After incubation with the secondary antibodies, the sections were sealed with DAPI Fluoromount-G (Southernbiotech, Birmingham, AL, USA). The images were acquired via fluorescence microscopy (LSM710, Zeiss, Germany). The images in each figure were presented both as single-color stain and the merged image. Thus, the localization of two markers on similar and different cells can be visualized. Cells that co-expressed CD14 (green) and CD16 (red) markers in a similar location were yellow. For quantification of cells in histologic sections, up to 3 pictures of each section of thyroid tissues were taken. The results were shown as the counts of positive cells per high power field (HPF).

### Statistical analysis

All statistical analyses were performed using IBM SPSS Statistics for Windows, version 19.0. *P* < 0.05 were considered statistically significant. Descriptive data are presented as mean ± SD. Differences between groups were calculated using t-tests or Mann–Whitney U tests. Correlations among the different variables were analyzed via Pearson’s or Spearman’s correlation analysis.

### Compliance with ethical standards

All procedures performed in studies involving human participants were in accordance with the ethical standards of review board of Ruijin Hospital affiliated to Shanghai Jiao Tong University Medical School and with the 1964 Helsinki declaration and its later amendments or comparable ethical standards. Written informed consent was obtained from each participant.

## Results

### Percentages and absolute counts of monocytes and correlations with clinical parameters in patients with GD

A total of 83 uGD patients and 30 healthy controls were analyzed in our study. A total of 72.3% uGD patients were females (n = 60), and 27.7% of the uGD patients were males (n = 23). uGD patients had increased FT3 and FT4 levels and a decreased TSH level in the serum, whereas thyroid function returned to normal in eGD patients and nGD patients were negative for TRAbs after antithyroid drug treatment (Table 1). Both the absolute counts and the percentages of monocytes in patients with uGD were significantly higher than those in HCs (Table 1, all *P* < 0.001). Under antithyroid drug treatment, the percentages and absolute counts of monocytes gradually decreased in patients with eGD or nGD and were not significantly different from those in HCs. Correlation analysis showed that both the absolute counts and percentages of monocytes were positively correlated with TRAbs in uGD patients (Table 2; *r*_*counts*_ = 0.269, *P* < 0.001; *r*_*percentage*_ = 0.338, *P* < 0.001). The absolute counts and percentage of monocytes were positively correlated with FT3 and FT4 and inversely correlated with TSH in patients with GD (Table 2).

**Table 1:** General characteristics of the study population.

Variables	HC	uGD	eGD	nGD
N (F/M)	30(20/10)	83(60/23)	83(60/23)	83(60/23)
Age (years)	31.68 ± 5.83	36.54 ± 12.76	38.42 ± 12.79	40.33 ± 11.87^d^
FT3 (pmol/L)	4.51 ± 0.45^b^	25.54 ± 14.01	4.45 ± 0.76^b^	4.19 ± 0.45^b^
FT4 (pmol/L)	13.56 ± 1.27^b^	36.79 ± 12.66	10.95 ± 2.62^b^	13.44 ± 1.82^b^
TSH (mIU/L)	1.88 ± 0.79^b^	0.0076 ± 0.093	1.91 ± 4.35^b^	1.91 ± 1.08^b^
TRAb (IU/L)	0.51 ± 0.20^b^	16.46 ± 12.95^a^	11.97 ± 11.77^ab^	0.66 ± 0.26^bc^
WBC (× 10^9^/L)	6.30 ± 1.34	6.17 ± 2.76	6.34 ± 1.69	6.76 ± 1.95
N%	59.30 ± 6.46^b^	52.63 ± 10.97	56.67 ± 8.98^b^	58.11 ± 11.99^b^
L%	32.42 ± 6.24	34.79 ± 9.23	33.89 ± 7.75	32.17 ± 8.39
M%	5.58 ± 1.38^b^	10.56 ± 6.6^a^	6.74 ± 1.92^b^	6.23 ± 1.73^b^
N (× 10^9^/L)	3.74 ± 0.88	3.39 ± 1.81	3.86 ± 3.45^b^	4.07 ± 1.52^b^
L (× 10^9^/L)	2.03 ± 0.52	2.10 ± 0.70	2.11 ± 0.63	2.11 ± 0.58
M (× 10^9^/L)	0.36 ± 0.13 ^b^	0.62 ± 0.28^a^	0.45 ± 0.41^b^	0.43 ± 0.15^b^

*HC* healthy controls, *uGD* untreated GD, *eGD* euthyroid GD, *nGD* TRAb-negative GD, *FT3* free triiodothyronine, *FT4* free thyroxine, *TSH* thyroid stimulating hormone, *TRAb* thyrotropin receptor antibody.

Compared to HC, ^a^*P* < 0.001; compared with uGD, ^b^*P* < 0.001; compared with eGD, ^c^*P* < 0.001; compared to HC, ^d^*P* < 0.05; *P* value was determined by Mann–Whitney U test.

**Table 2:** The correlation between monocytes and thyroid function parameter and antibody to thyrotropin receptor (TRAb) in Graves’ disease.

Parameters	Percentage of monocytes		Absolute count of monocytes	
	*r*	*P*	*r*	*P*
FT3	0.372	*P* < 0.001	0.285	*P* < 0.001
FT4	0.342	*P* < 0.001	0.283	*P* < 0.001
TSH	− 0.142	*P* < 0.001	− 0.143	*P* < 0.001
TRAb	0.338	*P* < 0.001	0.269	*P* < 0.001

Correlations were analyzed via Spearman’s correlation analysis.

*FT3* free triiodothyronine, *FT4* free thyroxine, *TSH *thyroid stimulating hormone, *TRAb* thyrotropin receptor antibody.

### Increased BAFF expression in monocytes and its correlations with clinical parameters in GD

**Figure 1:**
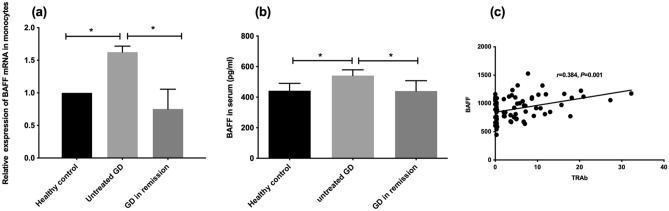
B cell activating factor (BAFF) expression in monocytes and serum and its correlation with TRAb. **(a)**, *BAFF mRNA* expression in the monocytes of healthy controls (n = 10), patients with untreated GD (n = 24) and patients with negative TRAb GD in remission (n = 6) using real-time PCR; **(b)**, ELISA assay for BAFF expression in the serum of healthy controls (n = 14), patients with untreated GD (n = 56) and GD patients in remission (n = 12); **(c)**, correlation analysis of TRAb and serum BAFF levels in patients with untreated GD; **P < 0.001.

Both the absolute counts and percentages of monocytes were strongly correlated with TRAbs in GD patients. We analyzed the expression of BAFF which was mostly secreted by monocytes and is involved in promoting the production of antibodies in B cells. We observed significantly elevated *BAFF mRNA* levels in monocytes from patients with uGD and those from HCs (Fig. 1a, P < 0.05). *BAFF mRNA* levels recovered to normal in patients with nGD (*P* < 0.05). The serum BAFF levels in individuals with uGD were significantly higher than those in HCs and of nGD patients (Fig. 1b, both *P* < 0.05). In addition, serum BAFF levels were positively correlated with TRAb levels (Fig. 1c, r = 0.384 and *P* = 0.001).

### Monocyte subsets in the peripheral blood of patients with GD and healthy controls

**Figure 2:**
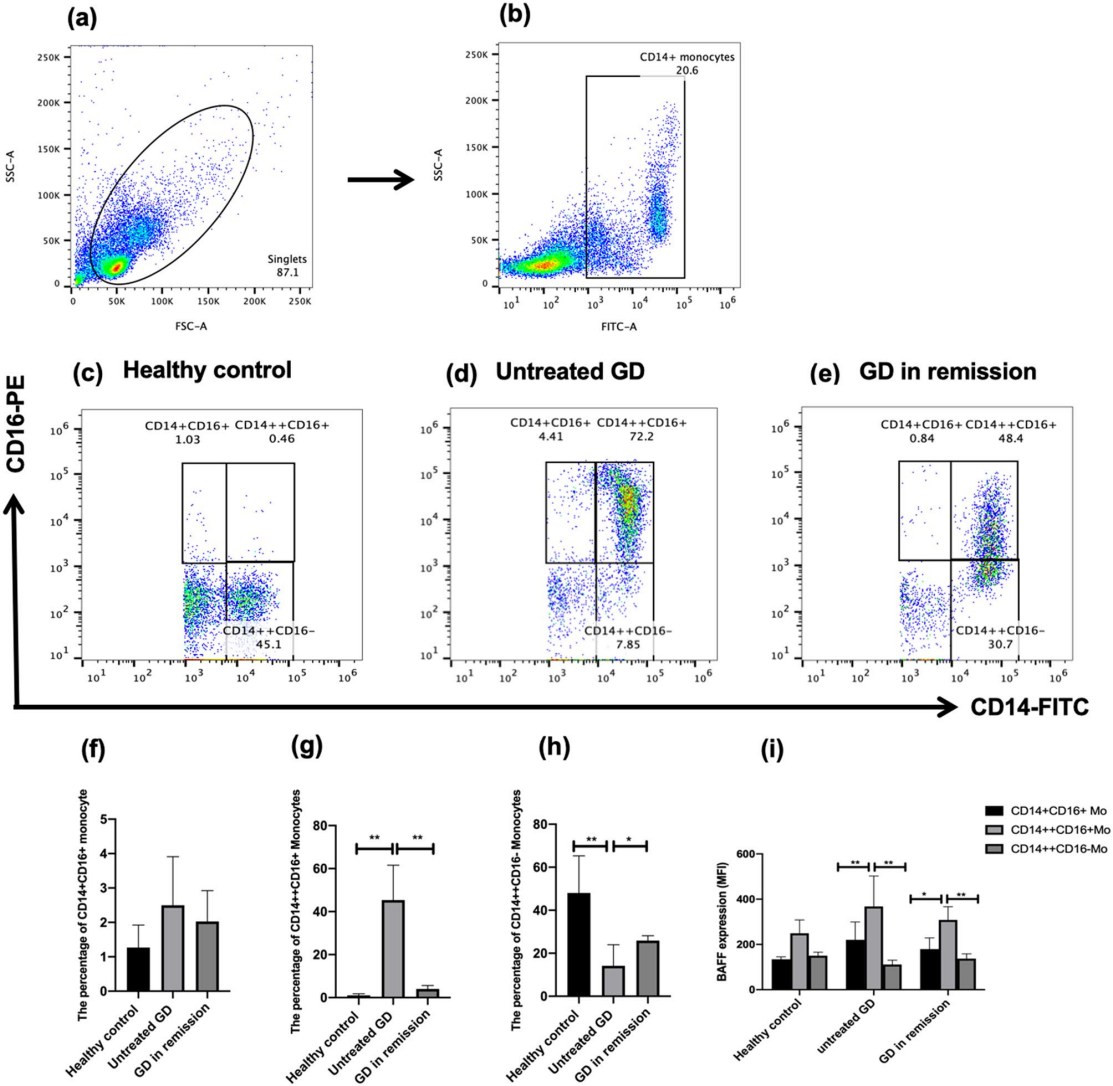
Characterization of the monocyte subsets in healthy controls and patients with Graves’ disease. **(a,b)** showed the gating strategy for monocytes, **(c–e)** representative dot plots of CD14 and CD16 expression on monocytes from healthy controls, untreated GD patients and GD patients in remission; **(f)** showed the percentages of CD14+ CD16+ monocytes (non-classical monocytes), **(g)** showed the percentages of CD14++ CD16+ monocytes (intermediate monocytes) and **(h)** showed the percentages of CD14++ CD16- monocytes (classical monocytes) from healthy control subjects (n = 10), untreated GD (n = 24) and negative TRAb GD in remission (n = 6), **(i)** showed the mean fluorescence intensity (MFI) of BAFF on different monocytes subsets; *P < 0.05, **P < 0.001.

Monocyte subsets were evaluated by flow cytometry. According to the expression of CD14 and CD16, the peripheral blood monocytes were classified into three subsets: CD14++ CD16+ , CD14+ CD16+ , and CD14++ CD16-. The gating strategy for the monocyte subsets is shown in Fig. 2a,b. CD14++ CD16+ monocytes were barely seen in HCs (Fig. 2c), CD14++ CD16+ monocytes were expanded in patients with uGD (Fig. 2d) and decreased in patients with nGD (Fig. 2e). The frequency of CD14+ CD16+ monocytes showed no significant difference among the three groups (Fig. 2f). The frequency of CD14++ CD16+ monocytes was significantly elevated in patients with uGD, while the frequency of CD14++ CD16+ monocytes was significantly recovered in patients with nGD (Fig. 2g, P < 0.001). Also, the frequency of CD14++ CD16- monocytes was significantly decreased in patients with uGD (Fig. 2h, P < 0.001).

Considering the elevated level of BAFF in the serum of patients with uGD, we investigated the expression patterns of BAFF in monocytes subsets. BAFF was expressed on monocytes in all of the 3 subsets. Flow cytometry showed that the MFI of BAFF on CD14++ CD16+ monocytes in uGD was significantly higher than that on CD14+ CD16+ monocytes and CD14++ CD16- monocytes (Fig. 2i, P < 0.05). Correlation analysis showed that the frequency of CD14++ CD16+ monocytes was positively correlated with the TRAb level (Table 3; *r* = 0.845 and *P* = 0.002).

**Table 3:** Correlation between TRAb and percentage of monocyte subsets.

	Nonclassical monocytes	Intermediate monocytes	Classical monocytes
	CD14+ CD16+ (%)	CD14++ CD16+ (%)	CD14++ CD16− (%)
*r*	0.562	0.845	− 0.687
*P*	0.091	0.002	0.500

Correlations were analyzed via Spearman’s correlation analysis.

### Monocytes infiltrated into the thyroid tissues of patients with GD

**Figure 3:**
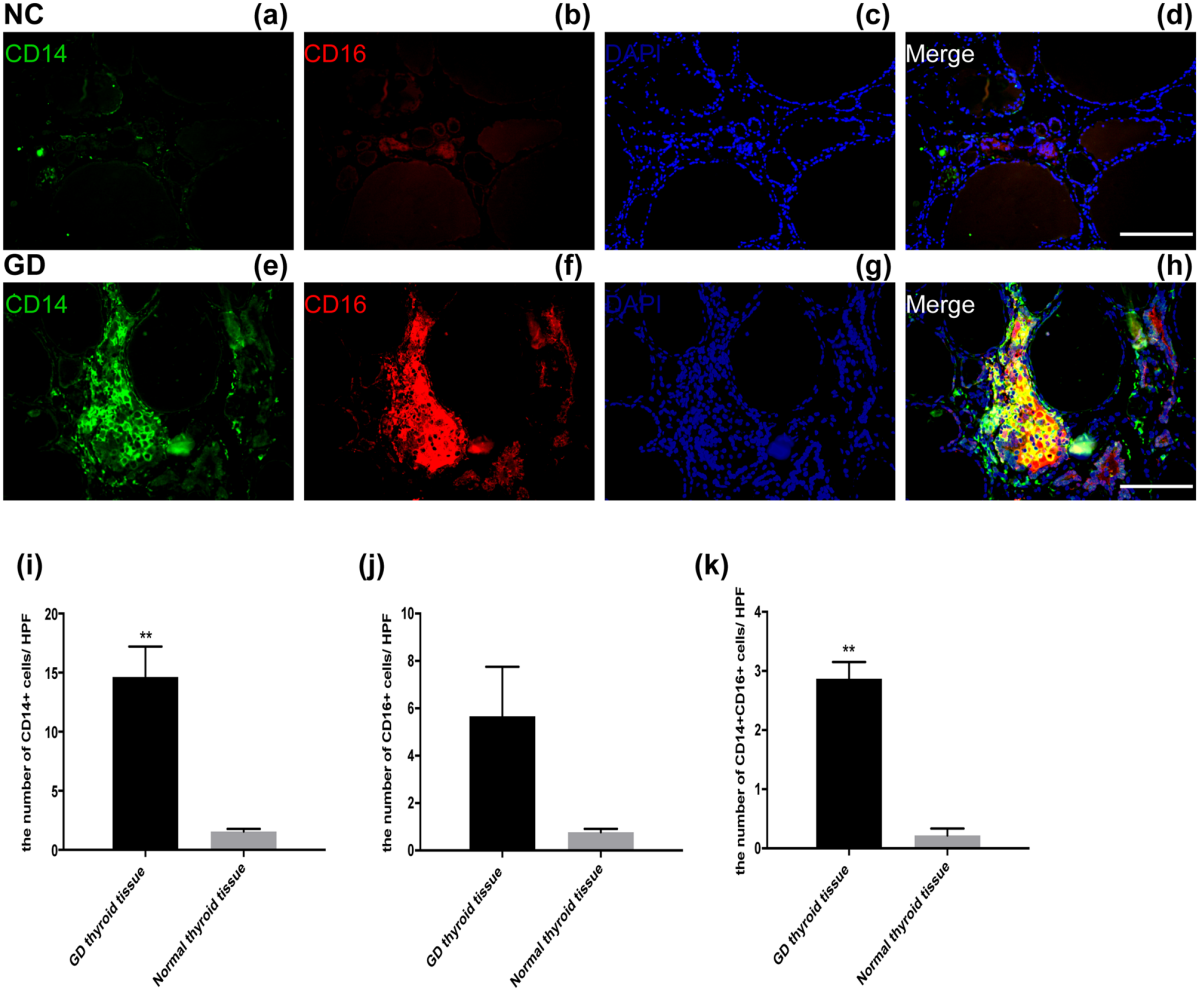
CD14 and CD16 expressing monocytes in the thyroid tissues of patients with Graves’ disease. Immunofluorescence staining was carried out using the samples of the normal thyroid tissues of patients with thyroid nodules **(a–d)** and patients with Graves’ disease **(e–h)**. The scale was 100 μm. Cells that co-express CD14 (green) and CD16 (red) markers in a similar location were yellow in color. The frequency of CD14+ , CD16+ and CD14+ CD16+ cells were calculated per high power field (HPF, × 400) in multiple samples: NC (n = 3, 15 sections) and GD (n = 6, 30 sections) were shown in **(i–k)**, respectively; **P < 0.001.

To investigate the presence of monocytes in thyroid tissue in GD, thyroid tissue samples were stained for CD14 and CD16. CD14+ , CD16+ , and CD14+ CD16+ cells were rarely observed in normal thyroid tissue (Fig. 3a–d). CD14+ , CD16+ and CD14+ CD16+ cells were found in the thyroid tissues of patients with GD, particularly between the structures of the thyroid follicles (Fig. 3e–h). The numbers of CD14+ cells, CD16+ cells and CD14+ CD16+ cells per high power field (HPF) were higher in GD thyroid tissue than in normal thyroid tissue (Fig. 3i–k; for CD14+ cells, *P* < 0.001; for CD14+ CD16+ cells, *P* < 0.001). The number of CD16+ cells per HPF was higher in GD thyroid tissue than normal thyroid tissue, although no statistical significance was found.

## Discussion

The present study evaluated the pathological role of monocytes in GD patients in Chinese adults. Monocytes are important cells of the innate immune system and play specific roles in the control, development and escalation of the immunological processes by presenting antigens and secreting specific cytokines^6–8^. Abnormal monocyte functioning may affect the development of inflammatory diseases. In our study, monocyte levels were significantly elevated in the peripheral blood of untreated GD patients. The strong correlation between the levels of monocytes and TRAbs suggested the putative pathological roles for monocytes in the production of TRAbs.

Monocytes are a heterogeneous population with phenotypical and functional differences. Based on the expression of CD14 and CD16 observed by flow cytometry, monocytes can be divided into three different subsets: CD14++ CD16- (classical monocytes), CD14++ CD16+ (intermediate monocytes), and CD14+ CD16+ monocytes (nonclassical monocytes)^7–8^. The expansion of intermediate monocytes has been observed in patients with autoimmune disease. Significantly elevated CD14+ CD16 ++ monocyte counts were found in patients with immunoglobulin A (IgA) nephropathy with normal renal function and blood cell counts^12^. Studies have demonstrated that monocytes are closely related to the clinical response in some autoimmune diseases. In untreated patients with rheumatoid arthritis, the detection of CD14++ CD16+ monocytes could predict the clinical response to methotrexate therapy^13^. Expanded monocytes might be a hallmark in relapsing–remitting multiple sclerosis and facilitate monitoring of the disease-modifying therapy response^14^. In our study, CD14++ CD16+ monocytes were significantly expanded in the peripheral blood in uGD patients, while CD14++ CD16+ monocytes were barely found in the peripheral blood in HCs. More importantly, positive correlations were observed between CD14++ CD16+ monocytes and TRAb level or thyroid function parameters in our study.

Further studies have been validated in functional studies that the intermediate monocytes had relatively higher antigen-presenting activity and a relatively higher proangiogenic capacity^20^. These monocytes are considered to be proinflammatory as they have an increased capacity to produce proinflammatory cytokines, such as TNF-α, IL-6, and IL-10 which were previously reported to be elevated in GD patients^1,3,6,21^. Secreted by monocytes, BAFF is a potent regulator of B cell development and functions, participating in promoting B-cell proliferation and antibody production^22–24^. In the present study, we found monocytes from patients with GD expressed a higher level of BAFF than those from HCs and that serum BAFF was positively correlated with TRAb levels. Although BAFF was expressed on monocytes in all 3 subsets, BAFF expression on CD14++ CD16+ monocytes was significantly higher than that on monocytes in the other two subsets. The results were in line with the existing literature. Mario et al. reported elevated serum BAFF concentrations in nine patients with GD and 43 patients with Graves’ ophthalmopathy^25^. Enhanced BAFF and BAFF-R expression in the infiltrating lymphocytes in GD thyroid tissue was also reported ^26^. It was proposed that new therapeutic approaches for GD patients should include B cell immunotherapy, such as administration of anti-CD20 antibody^27^. Animal evidence has shown that BAFF blockade reduces the occurrence of hyperthyroidism in murine models of GD^28^. In the clinic, BAFF antagonists have been developed and administered to treat autoimmune diseases such as rheumatoid arthritis^29–32^. Thus, our results indicated that monocytes may be involved in the production of TRAbs by secreting BAFF and that BAFF may become a new interventional target for B cell immunotherapy target in patients with GD in the future.

One of the contributions of monocytes to the pathogenesis of autoimmune disease is modulation of the adaptive immune system. More importantly, monocytes can migrate into locally inflamed tissue. In local tissue, monocytes may differentiate into macrophages and DCs to amplify the local immune response and antigen presentation. GD is an antibody-mediated organ-specific autoimmune disease. The immune microenvironment within thyroid tissue is critical for the production of TRAb. In our study, the frequencies of CD14+ monocytes and CD14+ CD16+ monocytes were significantly higher in GD thyroid tissue than that in normal thyroid tissue. Infiltrating CD14+ monocytes and CD14+ CD16+ monocytes were found to be infiltrated particularly between the structures of the thyroid follicles. The infiltration of T/B lymphocytes was validated in previous study. Hammerstad et al. reported an increase in CD8 + T cells in thyroid tissue during the chronic stage of GD and the presence of plasmacytoid dendritic cells (pDCs) at the lesions in thyroid tissue^33^. The presence of follicular helper T cells (Tfh cells) regulating the antibody production of B cells was also found in GD thyroid tissue^5^. Monocytes can activate CD4 + and CD8 + T cells via migration to the draining lymph nodes or stimulate effector and memory T cells directly at sites of inflammation^34^. In patients with rheumatoid arthritis, monocytes differentiate into inflammatory DCs in the synovial fluid and promote a Th17-mediated immune response via the secretion of IL-17^17^. Thus, the immune response within local thyroid tissue will be stimulated, maintained and amplified in a microenvironment composed of infiltrating T cells, B cells and monocytes. Monocytes infiltrating in GD thyroid tissue might participate in the cross-talk of T lymphocytes and B lymphocytes, promoting antibody production in the thyroid microenvironment.

The strength of our study was that we analyzed the monocytes in the periphery of patients with GD at different stages of disease and treatment. Our study is the first to investigate the monocyte subsets and identify the presence of monocytes in GD thyroid tissue. In addition, our study established a putative role for monocytes in the pathogenesis of GD. The limitation was that sample size was small and we failed to isolate the monocytes from thyroid tissue in GD patients which could help us to further investigate the pathological of monocytes in thyroid tissue.

## Conclusions

Collectively, our results revealed that CD14++ CD16+ monocytes were significantly increased in patients with untreated GD and might participate in the production of TRAbs by secreting relatively higher levels of BAFF. The presence of monocytes was identified in the thyroid tissues of patients with GD, which might maintain and amplify inflammatory responses in situ. Our study provides insights regarding the pathogenic roles of monocytes in the production of TRAbs in GD.

## Supplementary Information

Supplementary Information.
