# Supplementary material for: Expansion of inflammatory monocytes in periphery and infiltrated into thyroid tissue in Graves’ disease
Source: Sci Rep. 2021 Jun 29;11:13443. doi: 10.1038/s41598-021-92737-4 (PMC8242071; doi:10.1038/s41598-021-92737-4)
Supplement: Supplementary file 1 — Supplementary Information. [file 41598_2021_92737_MOESM1_ESM.docx]

**Expansion of inflammatory monocytes in periphery and infiltrated into thyroid tissue in Graves’ disease**

Xinxin Chen^1,2^, Yanqiu Wang^1^, Yicheng Qi^1^, Jiqi Yan^3^, Fengjiao Huang^1^, Mengxi Zhou^1^, Weiqing Wang^1^, Guang Ning^1^, Yulin Zhou^1^*, Shu Wang^1^*

1. Shanghai Clinical Center for Endocrine and Metabolic Diseases, Department of Endocrinology, Shanghai Institute of Endocrine and Metabolic Diseases, Ruijin Hospital, Shanghai Jiao Tong University Medical School, 197 Ruijin 2nd Road, Shanghai 200025 P.R. China.
2. Department of Endocrinology, Suzhou Municipal Hospital, Nanjing Medical University, 26 Daoqian Road, Suzhou 215002 P.R. China.
3. Department of Thyroid Surgery, Ruijin Hospital, Shanghai Jiao Tong University Medical School, 197 Ruijin 2nd Road, Shanghai 200025 P.R. China.

*: Corresponding author:

Yulin Zhou

Shanghai Clinical Center for Endocrine and Metabolic Diseases, Department of Endocrinology, Shanghai Institute of Endocrine and Metabolic Diseases, Ruijin Hospital, Shanghai Jiao Tong University Medical School

Address: 197 Ruijin 2nd Road, Shanghai 200025 P.R. China.

Email : yulinzhou6[@163.com](mailto:shuwangruijin999@163.com)

Tel: +86-021-64370045

Fax: +86-021-64333548

Shu Wang

Shanghai Clinical Center for Endocrine and Metabolic Diseases, Department of Endocrinology, Shanghai Institute of Endocrine and Metabolic Diseases, Ruijin Hospital, Shanghai Jiao Tong University Medical School

Address: 197 Ruijin 2nd Road, Shanghai 200025 P.R. China.

Email : [shuwangruijin999@163.com](mailto:shuwangruijin999@163.com)

Tel: +86-021-64370045

Fax: +86-021-64333548

Supplemental table S1. Primary Antibodies used for immunofluorescence

| Antigen | Primary antibodies | Manufacturer | Host | Iso | Dilution | Amplifaction/Detection |
| --- | --- | --- | --- | --- | --- | --- |
| CD14 | Anti-CD14[1H5D8] | Abcam | mouse | IgG | 1:200-1:1000 | Goat Anti-Mouse IgG H&L (Alexa Fluor 647) |
| CD16 | Anti-CD16[EPR4333] | Abcam | Rabbit | IgG | 1:100-1:250 | Goat Anti-Rabbit IgG H&L (Alexa Fluor 594) |


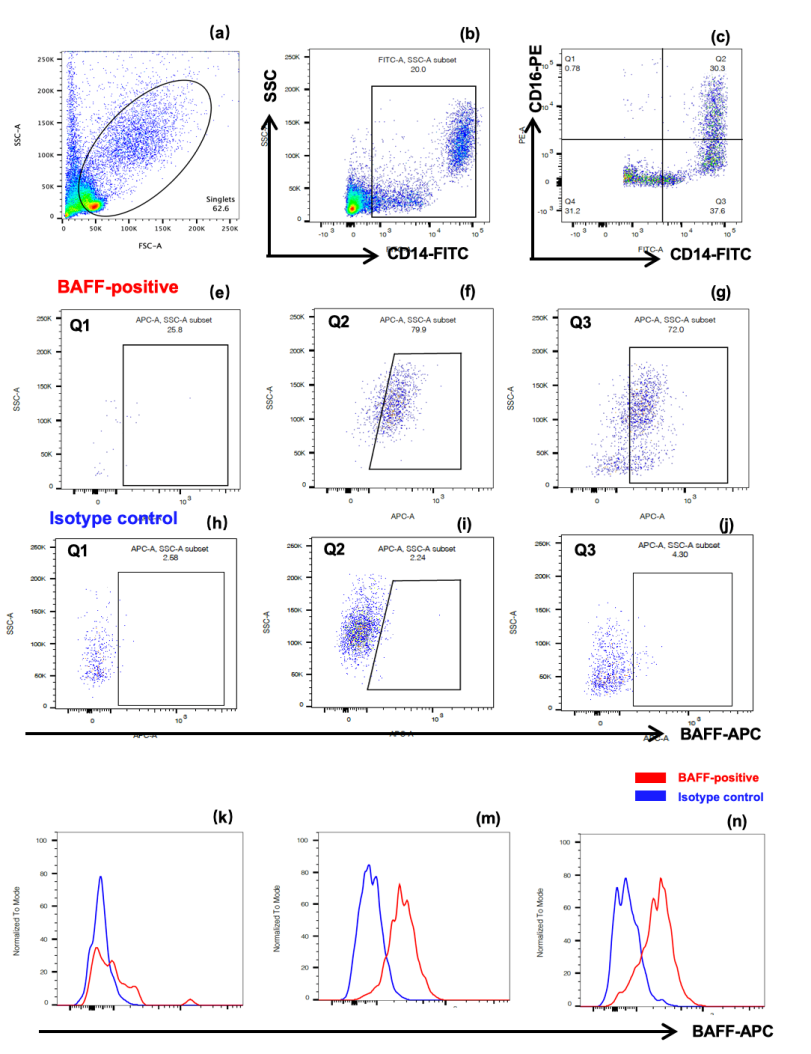


Supplemental figure S1. The isotype control for BAFF for 3 monocyte subpopulations

(a)~(c), showed the gating strategy for monocytes subpopulations; (e)~(g), showed representative dot plots of BAFF-APC expression on monocytes subpopulations; (h)-(j), showed representative dot plots of isotype control for BAFF labeled with APC; (k)-(n) showed the mean fluorescence intensity (MFI) of BAFF or isotype control on different monocytes subsets.
